# Urobiome Analysis in Interstitial Cystitis/Bladder Pain Syndrome Reveals Nuanced Differences Associated with Localized Pain

**DOI:** 10.1007/s00192-026-06716-2

**Published:** 2026-06-20

**Authors:** Seth A. Reasoner, Jamisha Francis, Mollie Gidney, Afia Amponsah, Brendan Frainey, Andrew Schrepf, A. Grace Kelly, Anna M. Ryden, Leslie J. Crofford, Roger R. Dmochowski, Maria Hadjifrangiskou, Lindsey C. McKernan

**Affiliations:** 1 Division of Molecular Pathogenesis, Department of Pathology, Microbiology & Immunology, Vanderbilt University Medical Center, Nashville, TN USA; 2 Department of Urology, Vanderbilt University Medical Center, Nashville, TN USA; 3 Department of Psychiatry and Behavioral Sciences, Vanderbilt University Medical Center, Nashville, TN USA; 4 Department of Anesthesiology, University of Michigan, Ann Arbor, MI USA; 5 Department of Medicine, Vanderbilt University Medical Center, Nashville, TN USA

**Keywords:** Urobiome, Interstitial cystitis, Urinary microbiome

## Abstract

**Introduction and Hypothesis:**

Interstitial cystitis/bladder pain syndrome (IC/BPS) is a prevalent chronic pain syndrome associated with functional urinary disorders. IC/BPS symptoms can be localized to the pelvic-region or have co-occurring widespread pain. Importantly, response to treatment depends on pain localization phenotype. The etiology of IC/BPS remains elusive, and whether bacteria contribute to IC/BPS pathophysiology remains uncertain. We hypothesized that the urobiome does not differentiate phenotypic presentations of IC/BPS.

**Methods:**

We used urine samples collected from a longitudinal randomized controlled trial of psychotherapy for individuals with IC/BPS to study the association of the urobiome and IC/BPS symptoms over time. Individuals provided urine samples at baseline, posttreatment, and at 5 months. We performed a secondary analysis on urine samples applying 16S rRNA sequencing and assigned bacterial taxonomy to amplicon sequence variants (ASVs) to characterize the urobiome. We compared urobiome diversity and stability over time, their associations with IC/BPS symptoms, and relationships with pain localization.

**Results:**

As validation of this dataset, we noted a strong influence of menopausal status and recent urinary tract infection on the composition of the urobiome. We did not detect widespread differences in the urobiome that correlated with an individuals’ pain localization or severity. Instead, we observed specific bacterial sequences that were altered in abundance in relation to symptomatology, such as reduced abundance of a *Dialister* ASV in persons with localized pelvic pain.

**Conclusions:**

Together, this dataset advances our understanding of the urobiome in IC/BPS and sets the stage for future studies on the urobiome and IC/BPS symptoms.

**Supplementary Information:**

The online version contains supplementary material available at https://doi.org/10.1007/s00192-026-06716-2.

## Introduction

Interstitial cystitis/bladder pain syndrome (IC/BPS) is a chronic pain syndrome characterized by debilitating bladder pain. IC/BPS affects an estimated 4–12 million patients in the United States [1]. The characteristic bladder pain of IC/BPS often increases with bladder filling. Other symptoms of IC/BPS include urinary frequency, urgency, painful sexual intercourse, and generalized pelvic pain [2]. Symptoms can be persistent and also include at times unpredictable and significant exacerbations or “flares.” The severity of symptoms in IC/BPS is associated with detrimental effects to patients’ quality of life. Frequent urination and painful symptoms negatively impact social functioning. The pathophysiologic cause of IC/BPS remains uncertain.

Given the proposed inflammatory nature of IC/BPS, a microbial etiology for IC/BPS has long been sought [3]. Many of the earliest urinary microbiome studies focused on IC/BPS to explore potential microbial contributions to IC/BPS pathophysiology. Presently, it is widely accepted that IC/BPS is a noninfectious condition [4]. Nonetheless, the unclear etiology of IC/BPS makes the resident microbiota of the genitourinary tract—the urobiome—a worthwhile research avenue in IC/BPS. Similarly, there has been speculation about whether urinary tract infections (UTIs) may be associated with or trigger IC/BPS onset [5]. Many of the symptoms overlap between UTIs and IC/BPS, such as dysuria and urinary urgency. The temporal relationship between UTIs and IC/BPS symptom onset and/or exacerbations remains uncertain.

Several studies have investigated the resident microbiota of the bladder in IC/BPS [6–11]. Another study used bladder biopsy samples to identify tissue-resident bacteria in IC/BPS [9]. Comparison of the urobiome of IC/BPS patients to healthy controls has consistently shown reduced microbiome alpha-diversity (within sample diversity) in urine samples from IC/BPS patients [6, 11, 12]. Conflicting results exist regarding the abundance of *Lactobacillus* in IC/BPS relative to healthy controls [11, 12]. These discrepancies likely arise from differences in sequencing technologies used and incomplete resolution of species of *Lactobacillus*. Thus far, the urobiome has not revealed any conclusive association with the pathogenesis or symptomatology of IC/BPS [12].

Inconsistent findings from previous investigations may be related to both sample and patient characteristics. Recent and novel research conducted by the Multidisciplinary Approach to Chronic Pelvic Pain (MAPP I/II) network emphasizes different pain “phenotypes” in IC/BPS, including individuals with more widespread versus localized pain [13]. IC/BPS includes distinct phenotypes, with widespread pain associated with systemic symptoms such as fatigue, cognitive dysfunction, and emotional distress, in contrast to localized pain confined to the bladder or pelvis. Systemic immunologic activity has been associated with the widespread pain phenotype in IC/BPS [14]. It is possible that inconsistent and variable reports on the urobiome are related to a patient’s phenotypic presentation. It is also important to examine the microbiome over time, especially given the variability of symptom severity in IC/BPS. Given similar symptoms between IC/BPS symptom flares and UTIs, it is crucial to determine whether microbial changes are linked to symptom flares to prevent potentially unwarranted antibiotic usage.

Unlike prior largely cross-sectional IC/BPS urobiome studies, this work includes detailed clinical data and longitudinal urine samples from a psychotherapy clinical trial to assess urobiome stability over time. We examined how urobiome variation relates to IC/BPS symptom severity and pain localization. We utilized rigorous bioinformatic processing to overcome limitations of prior IC/BPS urobiome studies. The strength of our dataset is its longitudinal nature, including up to three urine samples from the same patients over a 6-month period which allowed us to quantify metrics of the stability of the urobiome. Multiple samples from the same individuals allowed us to disentangle intra-individual effects from cohort-wide associations.

## Methods

### Sample and Metadata Collection

Urine samples were collected from a subset of patients in a clinical trial evaluating cognitive behavior therapy versus active symptom monitoring for IC/BPS (NCT #04275297; [15]). Study procedures and sample collection were approved by the Medical Center IRB (IRB #193461). Eligibility was determined using the RAND Interstitial Cystitis Epidemiology (RICE) screening tool and patient self-reported IC/BPS diagnosis [1, 15]. Patients provided written informed consent. For this project, we analyzed a subset of study participants who underwent additional in-person assessment visits providing data on IC/BPS phenotypic characteristics, including evaluation of the urinary microbiome. In addition to completing patient-reported outcomes via Redcap, participants also completed in-person study visits pretreatment (*n* = 38), immediately posttreatment (*n* = 35), and at 3 months posttreatment (*n* = 30).

Clean catch midstream urine samples were collected at each in person study visit. Patients were instructed on periurethral sterilization prior to urine collection. Urine samples were immediately stored at −80 °C, a separate portion of urine was saved at room temperature for urinalysis. Samples were shipped on dry ice to University of California San Diego Microbiome Core for DNA extraction and sequencing.

Clinical data and symptoms were obtained from patient-reported outcomes, in-person assessments, and medical records. Menopausal status was based on reported last menstrual period. Recent UTI was based on the RICE questionnaire at enrollment indicating UTI or antibiotic use for UTI within the prior 3 months. One participant reported a UTI during the study and provided a urine sample while on antibiotics; their samples were included in the recent UTI analysis group (patient 113 follow-up, Additional file 1). Patient symptom severity and localization were determined using validated questionnaires, including the Genitourinary Pain Index (GUPI) and Widespread Pain Index [16, 17]. Complete deidentified sample metadata is included in Additional file 1.

### DNA Extraction and 16S rRNA Sequencing

DNA extraction and 16S rRNA sequencing was performed at University of San Diego Microbiome Core. DNA was extracted from 0.5 mL of urine using the MagMAX™ Microbiome Ultra Nucleic Acid Isolation Kit (A42357, ThermoFisher) automated on a KingFisher Flex. Eight extraction blanks were included per 96-well DNA extraction plate. Serial dilutions of the ZymoBIOMICS™ Microbial Community Standard (D6300, Zymo) were extracted in parallel with urine samples, one set per DNA extraction plate. The V4 region of the 16S rRNA gene was amplified using the 515 F and 806R standardized primers [18]. PCR no-template blanks were included. Paired-end 250 bp reads were sequenced using the Illumina MiSeq platform. Unfiltered read counts are depicted in Fig. S1a and Additional file 1.

### Processing of 16S rRNA Sequencing Data

Raw sequence files were processed in R (version 4.4.1) using DADA2. Taxonomy was assigned to amplicon sequence variants (ASVs) with the SILVA rRNA database (version 138.1). When possible, species level assignment was made with the assignSpecies DADA2 function. ASVs are identified by the first four letters of their genus taxonomic assignment and an ASV label based on total abundance (e.g., Lact_ASV1, Additional file 2).

Using the methodology established by KatharoSeq [19], we calculated the proportion of ASVs from the mock community serial dilutions that had the expected taxonomic composition. Allosteric sigmoidal modeling showed that samples with greater than 864 reads had > 50% the expected taxonomic composition (K_1/2_, Fig. S1b). We set this as the threshold above which we considered the sample to be positive by 16S rRNA sequencing. None of the extraction blanks had greater than this read threshold (Fig. S1a, Additional file 1), and 98.1% (*n* = 101/103) of the study’s samples had > 1000 reads. This read threshold corresponded to between 525 and 5250 bacterial cells of mock microbial standard added per well (Additional file 1). Urine samples had a median of 42,646 reads (interquartile range 25,016, 57,979, Additional file 1) before bioinformatic decontamination.

We next engaged in a process filtering ASVs in the dataset, consistent with analysis standards for low biomass samples. To this end, we used the statistical program Decontam to identify potential contaminants in urine samples [20], as low prevalence and abundance ASVs over-represented in negative controls can conceal true bacterial signal. Negative controls included 16 extraction blanks and four PCR blanks. Following the methodology of Karstens et. al, we benchmarked bioinformatic decontamination using the serial dilutions of a mock microbial community of known composition (Fig. S2) [21]. We tested various Decontam thresholds with the goal of reducing contaminating ASVs without removing the expected taxa. Consistent with prior research, we used a threshold of 0.5 using the prevalence method of Decontam [21]. Within the entire dataset, there were 1061 ASVs prior to decontamination, and 365 ASVs remained after decontamination. The filtered ASVs were typically low abundance within urine samples. The mean percentage of reads filtered from urine samples was 4.995% and the median was 0.57% (Fig. S1c).

### Statistical Analysis of 16S rRNA Sequencing Data

We evaluated differences in β-diversity for patient variables, including UTI history, menopausal status, urinalysis results, pain localization, and GUPI scores. β-diversity was evaluated by Bray–Curtis dissimilarity indexes which were calculated with the phyloseq package’s functions *ordinate* and *distance* at the ASV level. To evaluate β-diversity differences between patient metadata variables, permutational multivariate analysis of variance (PERMANOVA) were computed with the vegan function *adonis2*, and patients with missing metadata for the variable being tested were omitted. The model included recent UTI history and menopausal status as co-variates.

We evaluated associations between patient variables (including UTI history, menopausal status, pain localization, and GUPI scores) and specific bacterial taxa at the genus and ASV levels. Differential abundance testing of ASVs and genera was conducted with Microbiome Multivariable Association with Linear Models (MaAsLin2) [22]. ASVs present in at least 5% of samples (i.e., prevalence 0.05) were included; no minimum abundance was specified. Relative abundances were log transformed within MaAsLin2, and the linear “LM” model was used. Patient ID was included as a random effect in the model due to multiple samples from the same patient, and missing metadata were included as “NA.” We performed secondary analyses, including menopausal status and prior UTI as co-variates. *P* values were adjusted for multiple comparisons using the Benjamini–Hochberg correction, with corrected values reported as false discovery rates (FDR). We considered the MaAsLin2 default FDR < 0.25 as significant for this exploratory study. The FDR < 0.25 threshold is the documented significance cutoff for MaAsLin2, and therefore is aligned with the tool developer’s recommendations for analyzing high dimensional microbiome data. Further, the FDR < 0.25 threshold is consistently applied to exploratory microbiome studies to avoid missing potentially meaningful signals that guide future investigations.

To identify ASVs suitable for persistence vs. transience analysis, we first restricted the dataset to ASVs detected in at least five different patients; this step ensured sufficient cross-patient representation for estimating persistence frequency. No minimum abundance threshold was applied, resulting in 104 ASVs included in the analysis (Additional file 3). Persistence was classified as the presence of the ASV in 2/2, 2/3, or 3/3 samples from the same patient. Transience was defined as an ASV only being present in one of a patient’s samples. Persistence frequency for each ASV was the number of patients for whom the ASV was persistent divided by the number of patients with at least one occurrence of the ASV. We used the Wilcoxon rank-sum test to compare persistence groups, and a *P* value of < 0.05 was considered significant.

## Results

### Study Cohort and Urobiome Characteristics

**Fig. 1:**
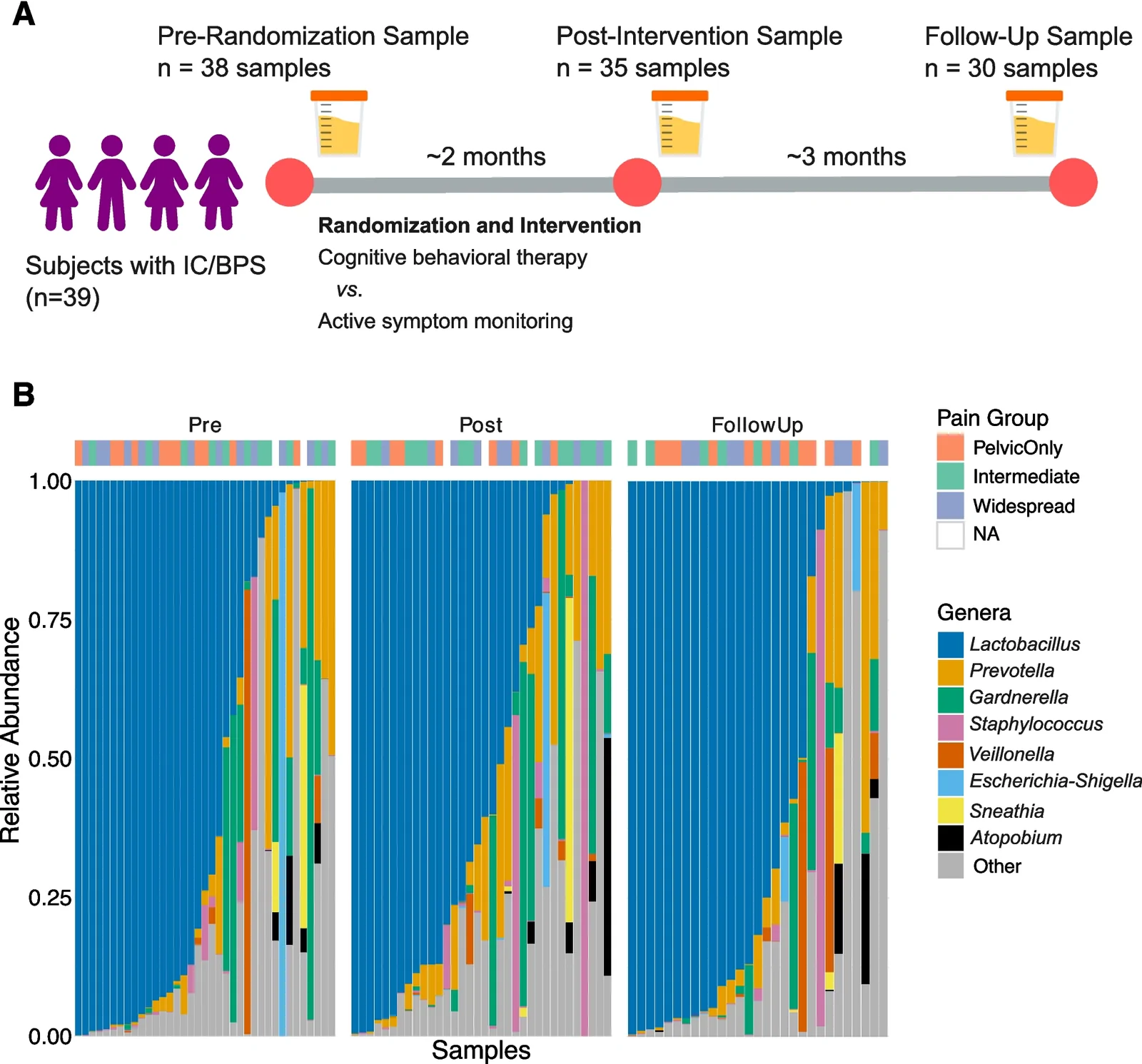
Interstitial cystitis: study schematic. **a** Urine samples were collected from patients undergoing a randomized control trial for cognitive behavioral therapy (CBT) for IC/BPS. Urine samples were collected at 3 timepoints throughout the study: before randomization (time = 0), after intervention (~ 2 months), and at follow-up (~ 5 months). **b** Relative abundance of bacterial genera in urine samples. The top 8 genera are depicted with samples ordered by *Lactobacillus* abundance. Pain localization metadata for each sample is depicted

Urine samples collected included 39 unique patients across three timepoints, totaling 103 urine samples (Fig. 1). Patients contributed an average of 2.64 samples (range 1–3 samples), with 27 patients (71%) completing all three timepoints. Within this cohort there were 37 women and two men (Table 1, Additional file 1). Over half of the women were premenopausal (56.8%), with two patients reporting diabetes and 11 patients reporting a recent UTI. Regarding IC/BPS symptoms on enrollment, 30.8% reported localized pelvic pain, and the mean total GUPI score was 27.5 (range 7–44) indicating moderate–severe pain symptoms, including a urinary sub-score mean of 6.3. A minority of participants had undergone bladder-directed procedures, including intravesical instillation (25.6%), hydrodistension (23.1%), and sacral nerve stimulation (10.3%) (Table 1). One patient had a documented Hunner’s lesions.

**Table 1:** Cohort characteristics

Participants (*n*)	39
Age, years (mean, [range])	44.8 [22–70]
Sex, *n* (%)	
Male Female	2 (5.1%) 37 (94.9%)
Characteristics	
Premenopausal, *n* (% of woman) diabetes, *n* (%) Recent UTI, *n* (%) Opioid prescription, *n* (%) Hunner’s lesions, *n* (%)	21 (56.8%) 2 (5.1%) 11 (28.2%) 5 (12.8%) 1 (2.6%)
Self-reported symptoms	
Localized pelvic pain, *n* (%) GUPI total score, mean (range) Urinary GUPI sub-score, mean (range)	12 (30.8%) 27.5 (7–44) 6.3 (1–10)
Self-reported prior treatments	
Intravesical installation, *n* (%) Hydrodistension, *n* (%) Sacral nerve stimulation, *n* (%)	10 (25.6%) 9 (23.1%) 4 (10.3%)

Clinical data obtained from patient self-report and/or medical record review. Data corresponds to the first sample from each patient in the study. Recent urinary tract infection (UTI) defined as self-reported UTI within 3 months of enrollment. Complete metadata is present in Additional file 1

Ninety-eight percent of urine samples (101/103) had > 864 16S rRNA reads and were classified as sequence positive. Sequencing results were bioinformatically decontaminated using Decontam (Fig. S2). The most abundant genera were *Lactobacillus* (median relative abundance, 72.4%; interquartile range [IQR], 6.4% to 94.9%), *Prevotella* (median, 1.8%; IQR, 0.05% to 11.6%), *Peptoniphilus* (median, 0.29%; IQR, 0% to 0.96%), and *Anaerococcus* (median, 0.24%; IQR, 0.02% to 1.0%) (Fig. 1b). The most prevalent genera were *Lactobacillus* (94/101 samples), *Prevotella* (82/101 samples), *Anaerococcus* (77/101 samples), and *Finegoldia* (77/101 samples). Urinary pathogens such as the genera *Escherichia* and *Klebsiella* were infrequent in the dataset (present in 20 and 7 samples, respectively).

**Fig. 2:**
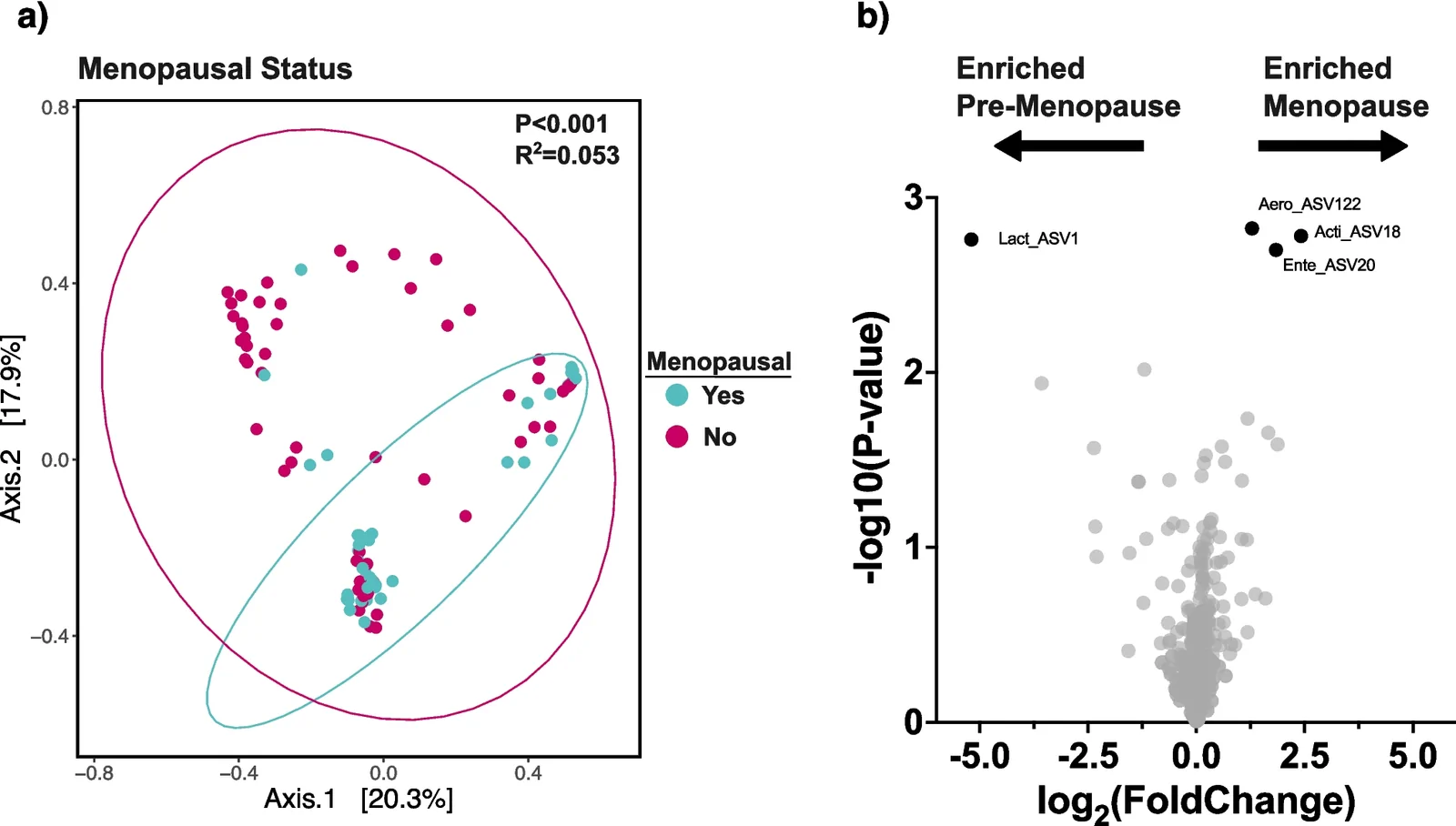
Menopause significantly impacts urobiome composition in IC/BPS. **a** Principal component analysis of Bray–Curtis distances of samples stratified by menopausal status. *P* value calculated by PERMANOVA. **b** Volcano plot of ASVs with respect to menopausal status. Differential abundance analysis conducted with MaAsLin2. Black labeled dots were significantly differentially abundant (MaAsLin2 FDR < 0.25) and correspond to the genera *Lactobacillus*, *Actinotignum*, *Enterococcus*, and *Aerococcus*

We first sought to evaluate the internal validity of our dataset. We validated expected differences in urobiome composition and diversity. We observed significant differences in β-diversity (a metric of sample similarity or dissimilarity) between samples from premenopausal and postmenopausal women in the cohort (Fig. 2a, PERMANOVA < 0.001, R^2^ = 0.053). Differential abundance analysis showed an increased abundance of a *Lactobacillus* ASV (Lact_ASV1) in premenopausal patients, whereas postmenopausal patients had an increased abundance of an *Actinotignum* ASV (Acti_ASV18), an *Enterococcus* ASV (Ente_ASV22), and an *Aerococcus* ASV (Aero_ASV122) (FDR < 0.25, Fig. 2b). Lact_ASV1 was assigned as *Lactobacillus iners*. When aggregated at the genus level, *Lactobacillus* was not differentially abundant with respect to menopause, suggesting that *L. iners* (Lact_ASV1) was reduced in menopause but other *Lactobacilli* were not uniformly reduced, thus emphasizing the importance of granular taxonomic analysis when possible. Other *Lactobacilli* species are not differentiable by sequencing of the V4 region of the 16S rRNA gene. Following menopause, a reduction in Lactobacillus in both the urinary and vaginal microbiota has been well documented, alongside reported increases in *Aerococcus* and a high prevalence of *Enterococcus* [23, 24].

**Fig. 3:**
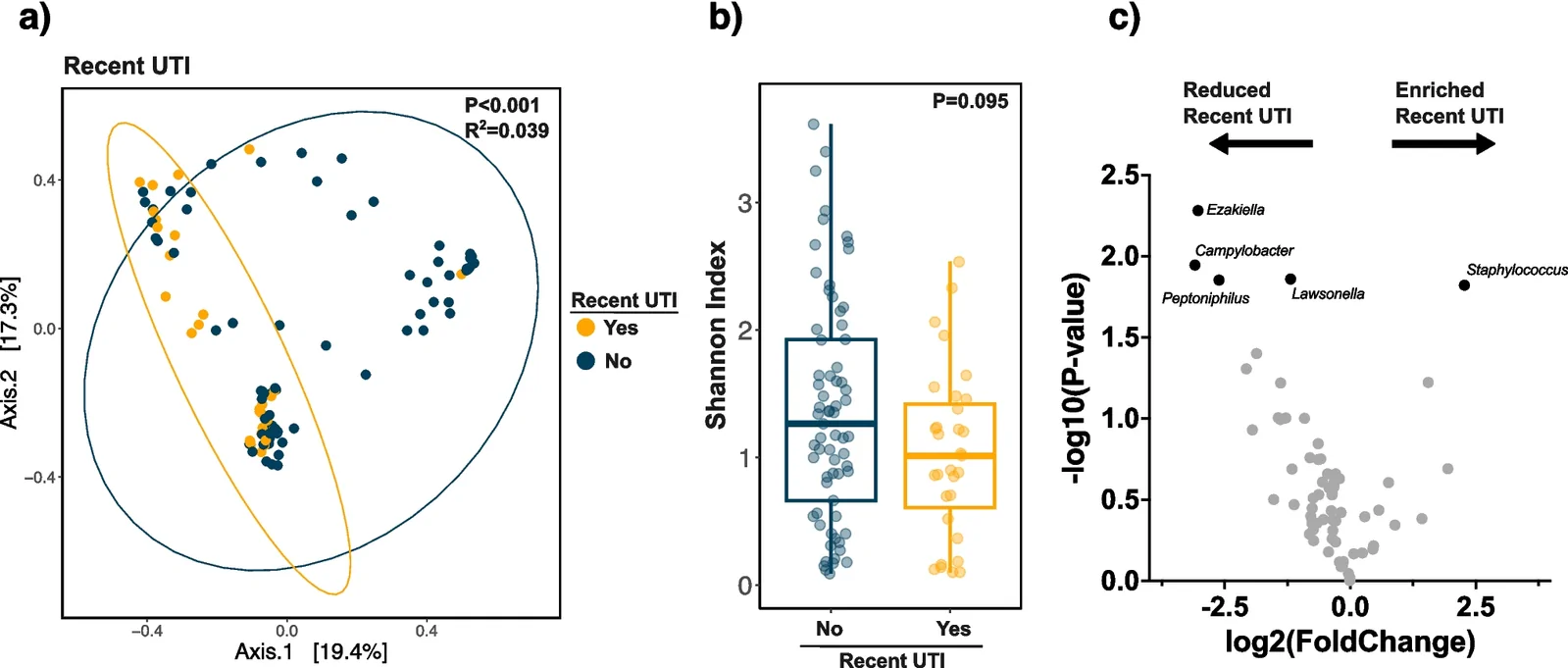
Recent UTI impacts urobiome composition in IC/BPS. **a** Principal component analysis of Bray–Curtis distances of samples stratified by recent UTI as defined by self-reported UTI within 3 months of study enrollment. *P* value calculated by PERMANOVA. **b** Shannon alpha diversity index of samples stratified by recent UTI. *P* values calculated by Wilcoxon rank-sum test. **c** Volcano plot of genera with respect to recent UTI. Differential abundance analysis conducted with MaAsLin2. Black labeled dots were significantly differentially abundant (MaAsLin2 FDR < 0.25). As noted in the main text, no individual ASV was differentially abundant, hence the plot displays differentially abundant genera

Urine samples from patients who reported a urinary tract infection (UTI) within three months prior to enrollment had significantly different β-diversity from patients without recent UTI (Fig. 3a, PERMANOVA < 0.001, R^2^ = 0.039). Recent UTI was associated with nominally reduced Shannon index, a measure of alpha diversity (*P* = 0.095, Fig. 3b). Differential abundance analysis did not show any differentially abundant ASVs. At the genera level, five genera were differentially abundant with respect to recent UTI (MaAsLin2 FDR < 0.25, Fig. 3c). The genera *Peptoniphilus*, *Campylobacter*, *Lawsonella*, and *Ezakiella* were reduced in abundance in samples from patients with recent UTI, whereas *Staphylococcus* was increased in abundance in samples from patients with recent UTI. We also detected differences in the urobiome composition based on urinalysis results (Fig. S3). For example, a *Veillonella* ASV was associated with the presence of nitrites on urinalysis. Intriguingly, among urinary pathogens, only a *Klebsiella* ASV was associated with positive urinalysis results (Fig. S3).

### Association of the Urobiome with Patient Symptomatology

Per our analysis plan, we sought to evaluate the relationship between urinary microbiome and patient pain phenotype, pain severity, and urinary symptom severity. Informed by previous investigations [25], we used the widespread pain index (WPI) to assess associations between pain phenotype and the urobiome, classifying patients as pelvic-only (“localized”), intermediate, or widespread pain, with the latter two grouped as “non-localized” pain [17].

**Fig. 4:**
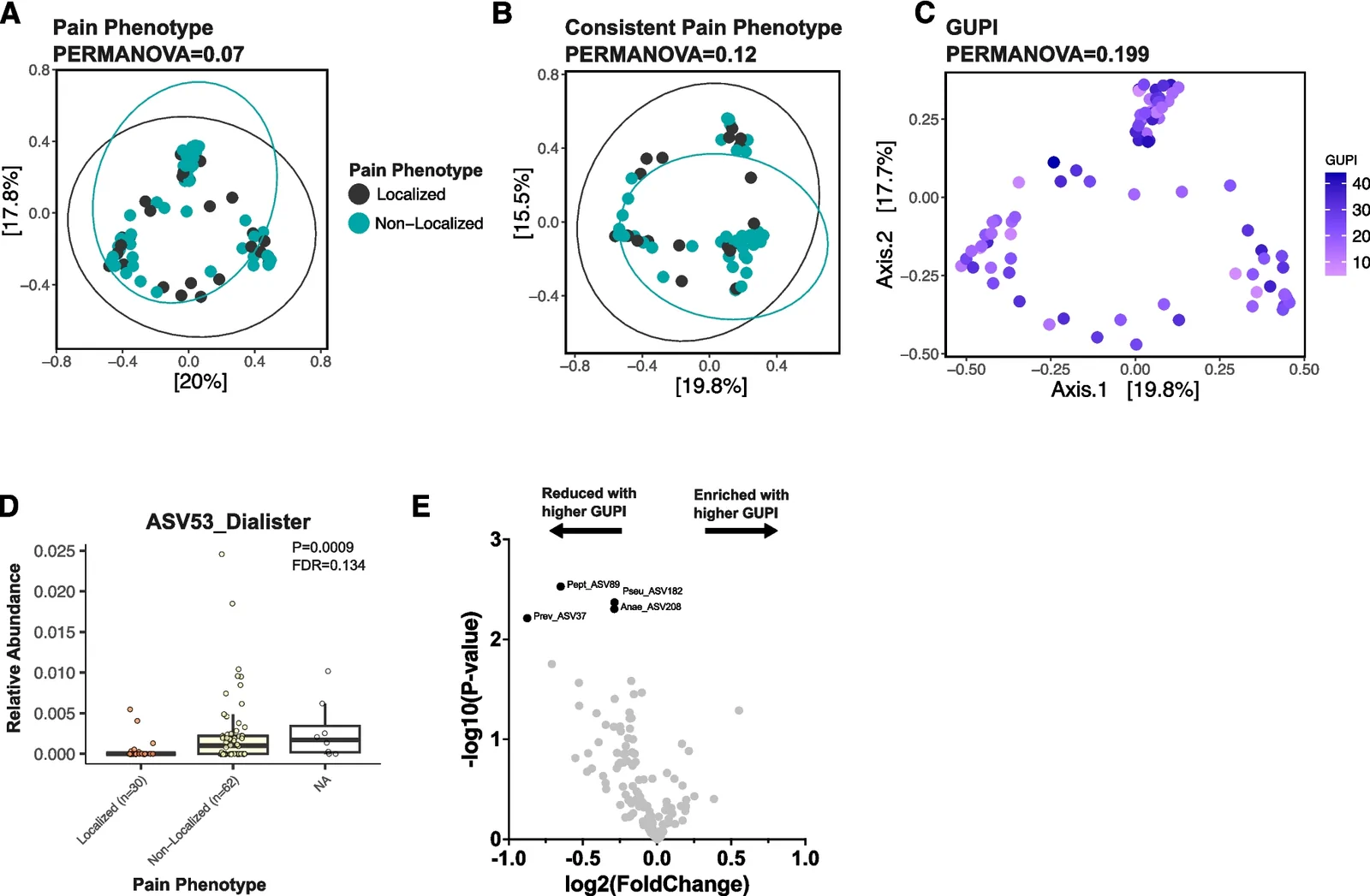
Association of patient symptomatology with the urobiome. **a** Principal component analysis of Bray–Curtis distances of samples stratified by pain localization phenotype. *P* value calculated by PERMANOVA and accounting for co-variates recent UTI and menopausal status. **b** Principal component analysis of Bray–Curtis distances of samples from patients with a consistent pain localization across timepoints (*n* = 23 patients). Samples stratified by pain localization. *P* value calculated by PERMANOVA and accounting for co-variates recent UTI and menopausal status. **c** Principal component analysis of Bray–Curtis distances of samples stratified by Genitourinary Pain Index (GUPI). *P* value calculated by PERMANOVA and accounting for co-variates recent UTI and menopausal status. **d** Abundance of Dial_ASV53 with respect to pain localization. Differential abundance analysis conducted with MaAsLin2. **e** Volcano plot of ASVs with respect to overall GUPI score. Differential abundance analysis conducted with MaAsLin2. Black labeled dots were significantly differentially abundant (MaAsLin2 FDR < 0.25) and corresponded to the genera *Prevotella*, *Peptoniphilus*, *Pseudoglutamicibacter*, and *Anaerococcus*

We began by assessing whether symptom localization is associated with urobiome diversity or composition. Pain localization did not significantly influence overall microbiome composition (PERMANOVA = 0.07, Fig. 4a). Similarly, stratification of pain phenotype by localized, intermediate, and widespread did not exhibit significant differences in β-diversity (Fig. S4). Because some patients experienced both pelvic pain only and widespread pain at different time points, we further analyzed the subset of patients with a consistent pain phenotype across study time points (*n* = 23 patients). There was no difference in overall microbiome composition between patients with consistent pain phenotypes (PERMANOVA = 0.12, Fig. 4b). As with symptom localization, overall symptom severity from the Genitourinary Pain Index (GUPI) did not correlate with overall urobiome composition (PERMANOVA = 0.199, Fig. 4c).

We next sought to determine whether any specific bacterial taxa were differentially abundant with respect to IC/BPS pain localization or severity of symptoms. A single ASV from the genus *Dialister* (Dial_ASV53) was reduced in abundance in patients with a localized pelvic-only pain phenotype (MaAsLin2 FDR = 0.134, Fig. 4d). Dial_ASV53 remained significantly less abundant in pelvic-only pain when controlling for menopausal status. Dial_ASV53 was present in low relative abundance in all samples (Fig. 4d). Four ASVs corresponding to the genera *Prevotella*, *Peptoniphilus*, *Pseudoglutamicibacter*, and *Anaerococcus* were reduced in abundance with greater overall GUPI scores (ASV37, ASV89, ASV182, ASV208, all MaAsLin2 FDR < 0.25, Fig. 4e, Additional file 6). We also conducted differential abundance testing using the GUPI urinary sub-score. We noted that ASVs corresponding to *Prevotella*, *Dialister*, *Peptoniphilus*, and *Anaerococcus* (ASVs 37, 53, 89, and 208) were among the ten most differentially abundant with respect to the GUPI urinary sub-score, though none of these ASVs met the significance threshold (Additional file 7, FDR > 0.25). Similar differentially abundant taxa between pain phenotypes, overall GUPI score, and GUPI urinary sub-score may indicate a common pattern of bacterial taxa influencing symptomatology in IC/BPS. Importantly, none of the ASVs that were correlated with symptomatology were differentially abundant with respect to menopause status or recent UTI, suggesting that these differentially abundant ASVs are not being confounded by menopause or recent UTI.

### Stability of the Urobiome over Time

**Fig. 5:**
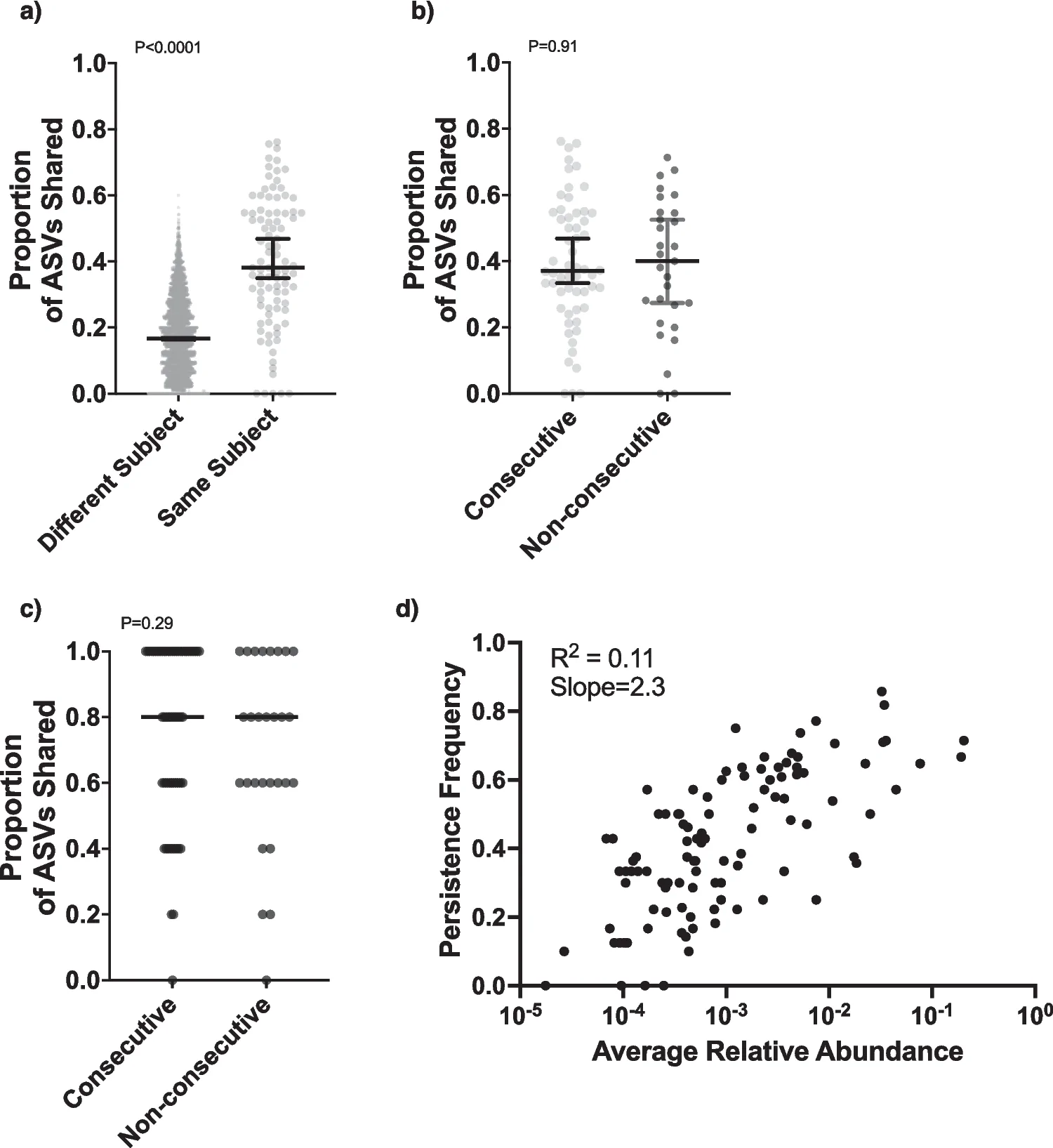
Urobiome stability across timepoints and within patients. **a** Proportion of shared ASVs between samples from the same or different patients. Error bars represent the median ± the 95% confidence interval. *P* value calculated by Wilcoxon rank-sum test. **b** Proportion of shared ASVs within the same patient at consecutive or nonconsecutive timepoints. Error bars represent the median ± the 95% confidence interval. *P* value calculated by Wilcoxon rank-sum test. **c** Proportion of the top five ASVs (sorted by mean relative abundance for each patient’s samples) shared between consecutive and nonconsecutive samples from the same patient. The horizontal line depicts the median. *P* value calculated by Wilcoxon rank-sum test. **d** Persistence frequency vs. average ASV abundance. R^2^ value determined by simple linear regression in GraphPad Prism v. 10.2.2

We evaluated the stability of the urobiome within individual patients. While 16S rRNA amplicon sequencing does not necessarily allow species or strain level taxonomic assignment, we used ASV comparison as a proxy for feature sharing between urine samples from the same patient. Across different patients, samples shared an average of 17% of ASVs, compared with 38% shared within the same patient over time (*P* < 0.0001; Fig. 5a). There were no differences between consecutive and nonconsecutive samples (Fig. 5b).

We speculated that more abundant ASVs would be more likely to be shared across timepoints. We extracted the top five ASVs from each patient’s samples by mean relative abundance and determined whether these ASVs were shared across timepoints. Indeed, among the top five ASVs from each patient’s samples, a median proportion of 0.8 were shared across timepoints (Fig. 5c). This proportion is double that of all ASVs shared within the same patient (0.8 vs. 0.38). In fact, the most abundant ASV from each patient was nearly universally present across timepoints from the same patient (*n* = 32/35 patients with samples from multiple timepoints).

Beyond the top five ASVs for each patient, we sought to determine which ASVs were persistent across samples from the same patient. We defined persistent ASVs as those present in > 50% of samples from a patient, and transient ASVs as those detected in only a single sample. Persistence frequency was calculated as the proportion of patients in whom an ASV was persistent among those for which at ASV appeared. Across all 104 ASVs included in the persistence analysis, the mean persistence frequency was 0.42. The most persistent ASV was a *Lactobacillus* ASV (Lact_ASV9) which was persistent in 12 of the 14 patients in whom the ASV was detected (0.86 persistence frequency). A *Staphylococcus* ASV (Stap_ASV5) was persistent in 27 of the 33 patients in whom it was detected (0.82 persistence frequency). Comparisons of persistence frequency may illuminate ecological insights about the role of specific taxa in the urobiome. For example, all seven *Lactobacillus* ASVs had a persistence frequency > 0.6, whereas ASVs assigned to the urinary pathogen genera *Escherichia* and *Enterococcus* had persistence frequencies < 0.4. We noted a moderate significant association between the persistence frequency and the average relative abundance of the particular ASV (R^2^ = 0.11, *P* = 0.0005, Fig. 5d). We interpret these data as representing that the more abundant taxa are stable across timepoints, whereas less abundant taxa are more likely to be transient.

## Discussion

Multiple factors may be contributing to IC/BPS etiology; contributing factors are thought to include urothelial dysfunction, chronic inflammation, psychosocial factors, and pelvic floor dysfunction. Whether microbes contribute to IC/BPS development or pathophysiology has been a perpetual question. Our results argue against a substantial microbial contribution to pain phenotypes or symptom severity in IC/BPS. That is, overall urobiome community composition as represented by β-diversity, as well as the abundance of the predominant bacterial groups, were not different between phenotypic groups (Figs. 1b and 4a). Instead, we detected specific low abundance bacterial taxa that were associated with pain localization and severity in IC/BPS. For example, an ASV corresponding to the *Dialister* genus (Dial_ASV53) was reduced in abundance among patients with a pelvic-only pain phenotype at a permissive FDR threshold (Fig. 4d). Intestinal abundance of *Dialister* has previously been identified to be depleted in patients with depression and negatively correlated with knee pain, suggesting a potential connection between this microbial taxa and pain [26, 27]. These taxa require validation in independent studies yet offer intriguing directions for future research regarding specific urobiome members and IC/BPS symptomatology. Together, these results suggest that large differences in urobiome composition do not determine IC/BPS symptomatology (Fig. 4a–c), whereas specific bacterial groups may correlate with symptom localization and severity. Few urobiome studies have considered the relationship between symptoms or pain localization and the urobiome [12]. Nickel et al. showed that IC/BPS patients had increased fungal members of the urobiome during symptoms flares [28]. Similarly, a study of patients female patients with Hunner’s lesions showed no differentiating urobiome features, while men with Hunner’s lesion had increased abundance of *Negativicoccus succinivorans*, *Porphyromonas somerae*, and *Mobiluncus curtisii* and decreased *Corynebacterium renale* [29]. These prior studies are consistent with our findings that substantial urobiome changes are not correlated with symptomatology, whereas the abundance of individual microbial members may be altered in abundance.

UTIs have been proposed as potential triggers of IC/BPS [5]. While our study cannot illuminate the causal relationship between UTIs and IC/BPS, we did detect a difference in urine samples from patients with and without self-reported recent UTI (Fig. 3a). The genera *Peptoniphilus*, *Campylobacter*, *Lawsonella*, and *Ezakiella* were increased in samples from patients with recent UTI, while *Staphylococcus* was decreased. Notably, these differentially abundant taxa are not canonical UTI pathogens, likely because participants were not actively experiencing UTI at the time of sample collection. Whether these differentially abundant taxa represent direct changes from UTI or are the indirect result of antibiotic treatment cannot be determined from this dataset. These differentially abundant taxa in recent UTI samples may inform future studies regarding urobiome members and UTI pathogenesis.

Since the vast majority of urobiome studies have been cross-sectional, little is known about the dynamics of the urobiome within individual patients. *Lactobacillus* ASVs were nearly twice as likely to be persistent compared to *Escherichia* and *Enterococcus* ASVs. Other urobiome studies have reported that *Lactobacilli* are stable within the same patient across timepoints [30]. While broad conclusions cannot be derived from this dataset, this approach offers a framework for determining metrics of urobiome stability.

This study has notable limitations. Only one patient in the study had documented Hunner’s lesions, limiting our ability to identify potential urobiome associations with these macroscopic bladder lesions. Similarly, the absence of healthy controls limits our ability to compare our findings from the IC/BPS urobiome to healthy individuals. Next, unlike prior urobiome studies of IC/BPS, our study had no healthy control population. Another limitation of this study is the lack of sampling controls, such as skin swabs or empty specimen cups, collected contemporaneously with the urine samples. We implemented negative controls throughout sample processing, such as DNA extraction and PCR amplification. The orders of magnitude difference in sequencing reads between urine samples and extraction controls (Fig. S1a) supports the authenticity of the bacterial signatures detected in urine samples. Similarly, we were able to detect differences in patient factors known to be associated with urobiome composition supporting the biological relevance of our sequencing results (i.e., menopause, recent UTI). Our use of 16S rRNA amplicon sequencing has inherent taxonomic limitations, including reduced resolution compared with culture and whole-genome sequencing approaches, but it remains well suited for amplifying bacterial DNA from low-biomass samples. Collection of voided urine also introduces potential contamination from periurethral or vaginal microbiota, which can confound interpretation of sequencing results. Future areas of investigation include replicating associations noted in this study, comparisons to healthy patients, and correlation of sequencing results with bacterial culture and will help refine the interpretation of the urobiome in IC/BPS.

In conclusion, we used longitudinally collected urine samples to study the urobiome and patient symptoms in IC/BPS. Widespread differences in urobiome diversity or composition were not associated patient symptom localization or severity. We detected reduced abundance of a *Dialister* ASV in patients with pelvic-only pain. Our future studies will attempt to independently replicate these findings and uncover the mechanisms underlying symptomatology in IC/BPS.

## Supplementary Information

Below is the link to the electronic supplementary material.

Fig. S1 Unfiltered read counts and KatharoSeq 16S rRNA read threshold. (**a**) Read counts of samples and blanks prior to bioinformatic decontamination. The box represents the 25th to 75th percentiles, while the whiskers represent the minimum and maximum read counts. Each data point is a unique sample. (**b**) The sequenced taxonomic composition of serial dilutions of the ZymoBIOMICS™ Microbial Community Standard was compared to the known taxonomic composition of the mock community standard. Allosteric sigmoidal non-linear regression was conducted in GraphPad Prism v. 10.2.2. (**c**) Proportion of 16S rRNA sequencing reads from urine samples retained following bioinformatic decontamination. Dark gray depicts retained reads and light gray depicts filtered reads (PDF 5383 KB)

Fig. S2 Bioinformatic decontamination of 16S rRNA sequencing from a mock microbial community. (**a**) Cell count of a ten-fold serial dilution of a mock microbial community. (**b**) Bacterial composition of the Zymo D6300 mock microbial community. (**c**) Read count of the mock microbial dilution prior to bioinformatic decontamination. (**d**) Read count of the mock microbial community after bioinformatic decontamination. (**e**) Stacked bar plot of sample composition before decontamination. (**f**) Stacked bar plot of sample composition after decontamination. For panels (**e**–**f**), the colored bars match expected taxa from the mock microbial community while the gray bars represent contaminants (PDF 5383 KB)

Fig. S3 Association of urinalysis results with urobiome composition. (**a**) Principal component analysis of Bray–Curtis distances of samples stratified by presence of urine nitrites. *P* value calculated by PERMANOVA. (**b**) Volcano plot of ASVs with respect to the presence of urine nitrites. Differential abundance analysis conducted with MaAsLin2. Black labeled dots were significantly differentially abundant (MaAsLin2 FDR < 0.25) and corresponded to the genera *Veillonella*, *Atopobium*, *Megasphaera*, *Klebsiella*, and *Corynebacterium* (PDF 5383 KB)

Additional file 1. Deidentified subject Metadata and unfiltered read counts. (XLSX 128 KB)

Additional file 2. ASV taxonomic assignment. (XLSX 54 KB)

Additional file 3. MaAsLin2 differential abundance with respect to menopausal status. (XLSX 26 KB)

Additional file 4. MaAsLin2 differential abundance with respect to recent UTI. (XLSX 26 KB)

Additional file 5. MaAsLin2 differential abundance with respect to pain localization phenotype. (XLSX 41 KB)

Additional file 6. MaAsLin2 differential abundance with respect to overall GUPI. (XLSX 26 KB)

Additional file 7. MaAsLin2 differential abundance with respect to urinary GUPI. (XLSX 26 KB)

Additional file 8. ASVs included in persistence analysis. (XLSX 15 KB)

Fig. S4 Association of urinalysis results with urobiome composition. **a**) Principal component analysis of Bray–Curtis distances of samples stratified by pain localization phenotype. *P* value calculated by PERMANOVA and accounting for recent UTI and menopausal status. **b**) Principal component analysis of Bray–Curtis distances of samples from patients with a consistent pain localization across timepoints (*n* = 16 patients). Samples stratified by pain localization. *P* value calculated by PERMANOVA and accounting for recent UTI and menopausal status. **c**) Abundance of Dial_ASV53 with respect to pain localization. Differential abundance analysis conducted with MaAsLin2 (PDF 5383 KB)

## Data Availability

Raw sequencing data is publicly available under NCBI BioProject PRJNA1141486. Bioinformatic code is available: https://github.com/reaset41/IC-Urobiome-Longitudinal.
