# Supplementary material for: Urobiome Analysis in Interstitial Cystitis/Bladder Pain Syndrome Reveals Nuanced Differences Associated with Localized Pain
Source: Int Urogynecol J. 2026 Jun 20;37(10):3585–95. doi: 10.1007/s00192-026-06716-2 (PMC13731717; doi:10.1007/s00192-026-06716-2)
Supplement: Supplementary file 1 [file 192_2026_6716_MOESM12_ESM.pdf]

Figure S1.

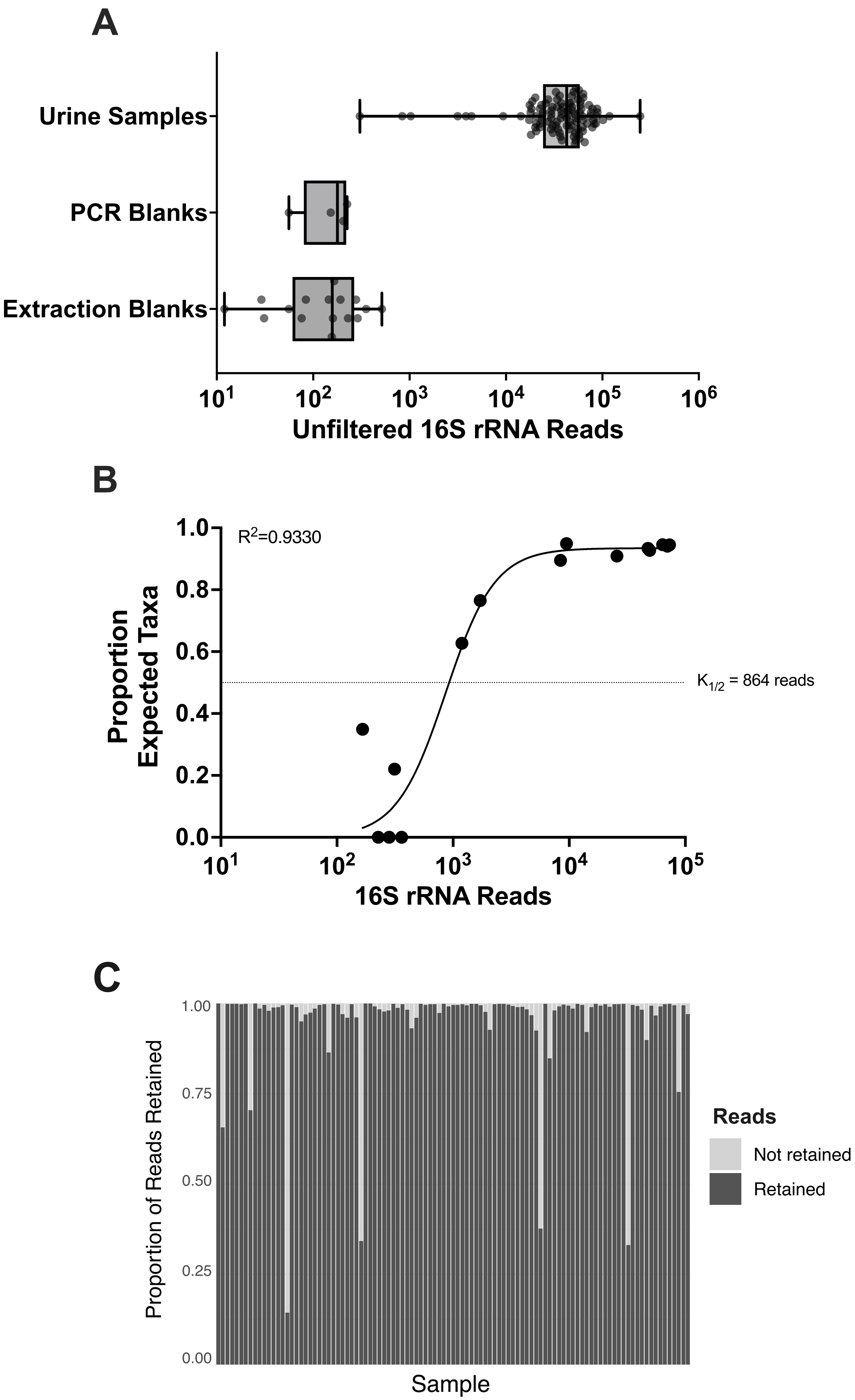
A
Urine Samples
PCR Blanks
Extraction Blanks
10^1 10^2 10^3 10^4 10^5 10^6
Unfiltered 16S rRNA Reads
B
R^2=0.9330
$K_{1/2}$ = 864 reads
Proportion Expected Taxa
1.0
0.8
0.6
0.4
0.2
0.0
10^1 10^2 10^3 10^4 10^5
16S rRNA Reads
C
Proportion of Reads Retained
1.00
0.75
0.50
0.25
0.00
Sample
Reads
Not retained
Retained
