# Supplementary material for: Urobiome Analysis in Interstitial Cystitis/Bladder Pain Syndrome Reveals Nuanced Differences Associated with Localized Pain
Source: Int Urogynecol J. 2026 Jun 20;37(10):3585–95. doi: 10.1007/s00192-026-06716-2 (PMC13731717; doi:10.1007/s00192-026-06716-2)
Supplement: Supplementary file 2 [file 192_2026_6716_MOESM1_ESM.pdf]

Figure S2.

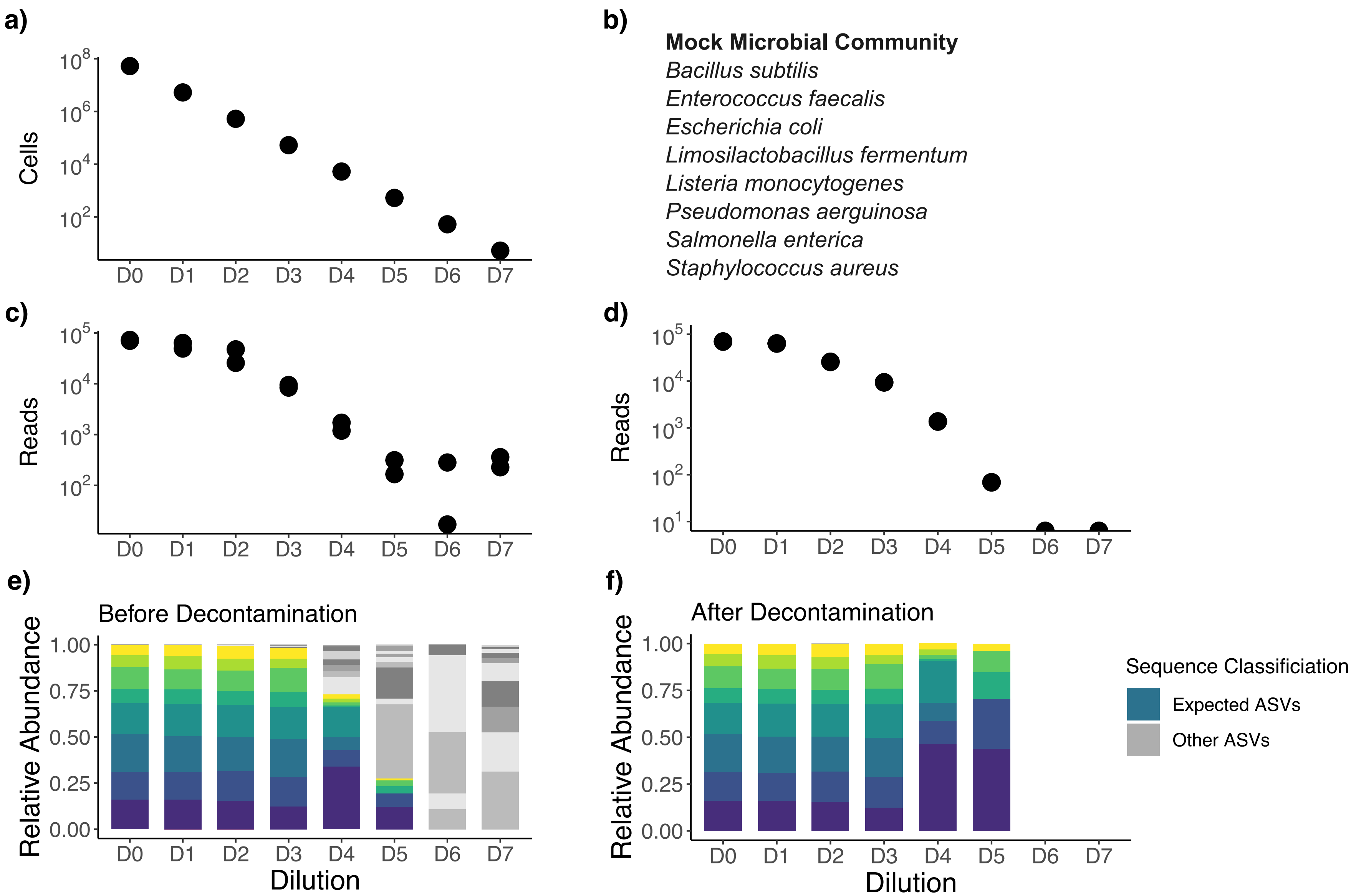

a)
Cells
10^8
10^6
10^4
10^2
D0 D1 D2 D3 D4 D5 D6 D7
b)
Mock Microbial Community
Bacillus subtilis
Enterococcus faecalis
Escherichia coli
Limosilactobacillus fermentum
Listeria monocytogenes
Pseudomonas aerguinosa
Salmonella enterica
Staphylococcus aureus
c)
Reads
10^5
10^4
10^3
10^2
D0 D1 D2 D3 D4 D5 D6 D7
d)
Reads
10^5
10^4
10^3
10^2
10^1
D0 D1 D2 D3 D4 D5 D6 D7
e)
Before Decontamination
Relative Abundance
1.00
0.75
0.50
0.25
0.00
D0 D1 D2 D3 D4 D5 D6 D7
Dilution
f)
After Decontamination
Relative Abundance
1.00
0.75
0.50
0.25
0.00
D0 D1 D2 D3 D4 D5 D6 D7
Dilution
Sequence Classificiation
Expected ASVs
Other ASVs
