# Supplementary material for: Urobiome Analysis in Interstitial Cystitis/Bladder Pain Syndrome Reveals Nuanced Differences Associated with Localized Pain
Source: Int Urogynecol J. 2026 Jun 20;37(10):3585–95. doi: 10.1007/s00192-026-06716-2 (PMC13731717; doi:10.1007/s00192-026-06716-2)
Supplement: Supplementary file 3 [file 192_2026_6716_MOESM2_ESM.pdf]

Figure S3.

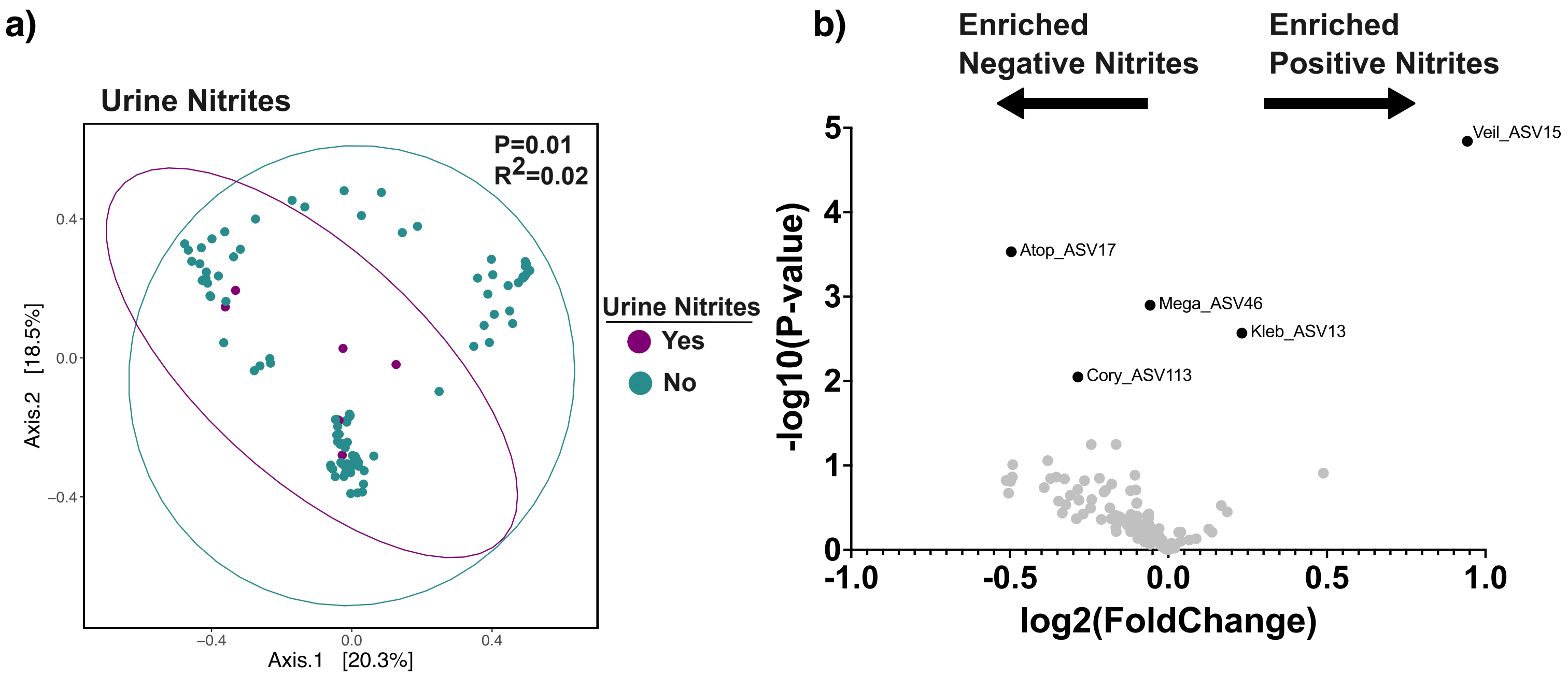

a)
Urine Nitrites
P=0.01
R2=0.02
0.4
0.0
−0.4
Axis.2 [18.5%]
−0.4
0.0
0.4
Axis.1 [20.3%]
Urine Nitrites
Yes
No
b)
Enriched
Negative Nitrites
Enriched
Positive Nitrites
Veil_ASV15
Atop_ASV17
Mega_ASV46
Kleb_ASV13
Cory_ASV113
5
4
3
2
1
0
-log10(P-value)
-1.0
-0.5
0.0
0.5
1.0
log2(FoldChange)
