# Supplementary material for: Urobiome Analysis in Interstitial Cystitis/Bladder Pain Syndrome Reveals Nuanced Differences Associated with Localized Pain
Source: Int Urogynecol J. 2026 Jun 20;37(10):3585–95. doi: 10.1007/s00192-026-06716-2 (PMC13731717; doi:10.1007/s00192-026-06716-2)
Supplement: Supplementary file 4 [file 192_2026_6716_MOESM3_ESM.pdf]

Figure S4.

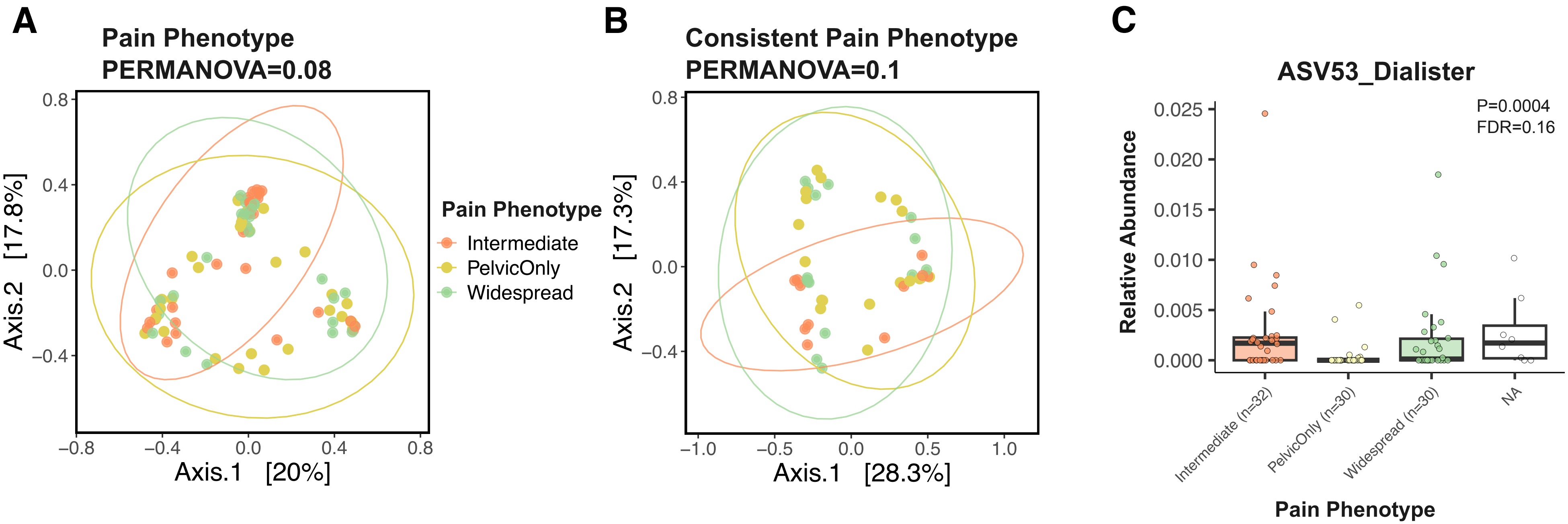
A
Pain Phenotype
PERMANOVA=0.08
Axis.1 [20%]
Axis.2 [17.8%]
Pain Phenotype
Intermediate
PelvicOnly
Widespread
B
Consistent Pain Phenotype
PERMANOVA=0.1
Axis.1 [28.3%]
Axis.2 [17.3%]
C
ASV53_Dialister
P=0.0004
FDR=0.16
Relative Abundance
Intermediate (n=32)
PelvicOnly (n=30)
Widespread (n=30)
NA
Pain Phenotype
